# Stress-induced loss of CTCF reveals an alternative, promoter-based mode of cohesin looping

**DOI:** 10.64898/2025.12.19.695003

**Published:** 2025-12-22

**Authors:** JP Flores, Andrea A. Perreault, Zack Drum, Chenxi Xu, Doris Cruz Alonso, Gelila Petros, Yijia Wu, Ivana Y. Quiroga-Barber, HyunAh Kim, Isha Sahasrabudhe, Justin Demmerle, Gang Greg Wang, Danfeng Cai, Douglas H. Phanstiel

**Affiliations:** 1 Curriculum in Bioinformatics & Computational Biology, Department of Genetics, University of North Carolina at Chapel Hill; 2 Department of Biology, Elon University, Elon, NC, 27244, USA; 3 Thurston Arthritis Research Center, University of North Carolina, Chapel Hill, NC 27599, USA; 4 Department of Pharmacology and Cancer Biology, Duke University School of Medicine, Durham, NC 27710, USA; 5 Department of Biochemistry and Molecular Biology, Johns Hopkins Bloomberg School of Public Health, Baltimore, MD 21205, USA; 6 Department of Oncology, Johns Hopkins School of Medicine, Baltimore, MD 21205, USA; 7 Department of Biophysics and Biophysical Chemistry, Johns Hopkins School of Medicine, Baltimore, MD 21205, USA; 8 Duke Cancer Institute, Duke University School of Medicine, Durham, NC 27710, USA; 9 Department of Pathology, Duke University School of Medicine, Durham, NC 27710, USA; 10 Department of Cell Biology and Physiology, University of North Carolina, Chapel Hill, NC 27599, USA

## Abstract

Cells continually encounter environmental stressors that challenge homeostasis. How three-dimensional (3D) chromatin structure contributes to these stress responses, particularly under hyperosmotic conditions, remains poorly understood. Here, using time-resolved Hi-C, CUT&Tag, auxin-inducible depletion, and RNA-seq, we map 3D chromatin structure, its molecular drivers, and transcriptional outcomes during the hyperosmotic stress response. Within 1 hour of sorbitol treatment, pre-existing loops and domains undergo genome-wide collapse, accompanied by the emergence of several hundred de novo, sorbitol-induced loops that are more punctate, longer-range, and transient. These newly formed loops weaken over time and largely dissipate by 24 hours, coincident with recovery of pre-existing chromatin structure. Loop reorganization is consistent across human cell types and hyperosmotic stimuli. CUT&Tag and degron experiments reveal that sorbitol-induced loops require cohesin but not CTCF. Newly formed loop anchors are enriched at active promoters containing SP and KLF family motifs. Genes located at these anchors show little immediate transcriptional change but are activated several hours after loop formation, consistent with loops functioning upstream of gene activation. Together, our findings show that hyperosmotic stress triggers a rapid, reversible, and CTCF-independent reorganization of 3D chromatin interactions that helps coordinate transcriptional adaptation.

Cells are constantly challenged by fluctuations in their environment, and their ability to mount rapid, coordinated responses to these stressors is critical for maintaining homeostasis and survival^1–5^. One common stressor encountered by human cells is rapid changes in osmolarity that must be adjusted to in order to preserve protein stability, membrane integrity, and nuclear organization^6–10,10–12^. In the kidney, for example, cells in the renal medulla experience high NaCl and urea and rely on osmoprotective programs to prevent damage, while in the immune system, elevated NaCl in the tumor microenvironment can enhance CD8^+^ T cell metabolic fitness and cytotoxicity^10,13,14^. Thus, hyperosmotic stress shapes cellular physiology in diverse tissues^9,10^ and understanding how cells adapt to osmotic stress could therefore provide broadly relevant insights into human biology and the mechanisms of gene regulation.

While much of the response to hyperosmotic stress has been defined through its transcriptional outputs^15–19^, the upstream layers of regulation—particularly those involving three-dimensional (3D) chromatin structure—remain incompletely understood. However, 3D chromatin structure plays an important role in gene regulation and mounting evidence suggests that the genome is rapidly remodeled when cells encounter hyperosmotic stress. We found that YAP1/TAZ, TEAD1, and accessible chromatin regions colocalize in human kidney cells under sorbitol-induced hyperosmotic stress, which suggests large-scale changes to 3D chromatin architecture^20^. Hyperosmotic stress has also been linked to redistribution of transcriptional co-regulators to accessible chromatin sites, weakening of chromatin domain boundaries, disruption of long-range looping interactions, widespread RNA polymerase II run-off and readthrough transcription, as well as large-scale chromatin condensation and compartment shifts^18,19,21–25^. In some systems, chromatin reorganization appears to be directed by specific architectural proteins rather than passive compaction alone^8,26^. Together, these studies suggest that hyperosmotic stress triggers coordinated structural and transcriptional remodeling of the genome.

Despite these findings, the connection between 3D chromatin structure and hyperosmotic stress response is not entirely clear. Many existing studies either did not directly map chromatin interactions, were performed in non-mammalian systems, or relied on shallow sequencing that could miss nuanced changes in 3D chromatin architecture^19–21^. As a result, the scope of 3D chromatin changes during hyperosmotic stress, the molecular machinery responsible, and the functional consequences for gene regulation remain incompletely defined.

To address these gaps, we mapped 3D chromatin structure, protein-DNA interactions, and gene expression in human cells responding to sorbitol-induced hyperosmotic stress. Together, we find rapid but transient remodeling of chromatin structure during hyperosmotic stress response and identify molecular features that may drive these structural changes. Our findings provide new insights into how cells reorganize their nuclear architecture under stress and open new avenues for exploring the interplay between 3D chromatin structure and transcription in health and disease.

## Results

### Hyperosmotic stress triggers widespread rewiring of chromatin loops

**Fig. 1 |:**
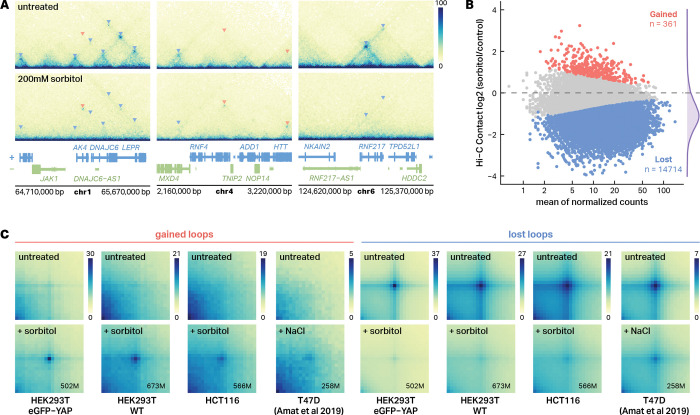
Hyperosmotic stress induces large-scale rewiring of chromatin interactions. **A**, Representative Hi-C contact maps showing chromatin structure changes at selected genomic loci in HEK293T eGFP-YAP1 cells. Top panels show untreated controls, bottom panels show cells treated with 200 mM sorbitol for 1 hour. Triangular indicators mark chromatin loops (red triangles = gained loops, blue triangles = lost loops). **B**, MA plot showing differential chromatin loop analysis between sorbitol-treated and untreated HEK293 cells. Red points indicate significantly gained loops (n = 361), blue points indicate significantly lost loops (n = 14,714). Statistical significance was determined by DESeq2 analysis (padj < 0.1, |log_2_FC| > 0 for gained loops, |log_2_FC| < 0 for lost loops). Density plot (purple) on the right show the distribution log2 fold changes. **C**, Aggregate Peak Analysis (APA) heatmaps demonstrating loop formation and loss across multiple cell lines and stimuli. Each heatmap shows aggregate contact frequency centered on loop anchors. Contact frequency is normalized by the max value for each cell type across treatment conditions. Treatment conditions: sorbitol (200 mM, 1h) for HEK293T eGFP-YAP1, HEK293T WT, and HCT116; NaCl (110 mM) for T47D. Average sequencing read depths (in millions) are indicated in the lower-right corner of each APA matrix.

To determine how hyperosmotic stress impacts 3D chromatin structure, we performed deeply sequenced Hi-C (8.18 billion total sequencing reads across 4 replicates per condition) in human embryonic kidney (HEK293T eGFP-YAP1) cells treated with 200 mM sorbitol for 1 hour, alongside untreated controls. Cells expressing eGFP-tagged YAP1 were used in order to follow up our recent studies mapping YAP1 phase separation in response to sorbitol, but as shown later, this modification did not influence results^20^. Hi-C contact maps revealed extensive structural changes after sorbitol treatment exemplified at loci shown in Figure 1A. The vast majority of pre-existing chromatin loops and chromatin structures were lost and were replaced via the formation of a smaller number of sorbitol-induced loops. Differential analysis revealed 14,714 lost and 361 sorbitol-induced loops after 1 hour of treatment (DESeq2, padj < 0.1) (Figure 1B). The global loss of pre-existing 3D chromatin structures has been observed in cells treated with 110mM NaCl^19^, but to the best of our knowledge, the gained loops in response to hyperosmotic stress had not been previously described. The vast majority (97.2%) of sorbitol-induced loops were formed *de novo* and were not observed in untreated cells, indicating a major reorganization of 3D contacts rather than strengthening of pre-existing loops (Figure S1). Notably, however, approximately half (49%) of sorbitol-induced loop anchors overlap with anchor positions present in untreated cells (Figure S1), suggesting that sorbitol-induced loops frequently repurpose existing architectural nodes.

To assess the generality of these effects, we performed Hi-C in both wild-type (WT) HEK293T cells and the colorectal cancer line, HCT116. To evaluate changes in looping across datasets, we performed aggregate peak analysis (APA) using the same set of loop anchor coordinates defined from the HEK293T eGFP-YAP1 differential loop calls as a shared reference. This approach enabled a direct, cell type-independent comparison of loop signal at a consistent set of genomic positions. In all three cell lines, APA showed strong depletion of pre-existing loop signal and the emergence of new loops following sorbitol treatment (Figure 1C). To determine if these results were due to sorbitol specifically or to hyperosmotic stress in general, we reanalyzed Hi-C data describing the treatment of the breast cancer cell line T47D with 110mM NaCl. While the initial paper describing those results did not report any gained loops, aggregate peak analysis revealed evidence of sorbitol-induced looping at the same regions where we observed gained loops in HEK293T WT, HEK293T eGFP-YAP1, and HCT116 cells. Weaker enrichment was observed in cells treated with NaCl than sorbitol, which could indicate differences in loop strength, loop locations, or the shallower sequencing of the NaCl-treated dataset (0.51 billion reads). Regardless, the persistence of loop enrichment across all conditions highlights the robustness of hyperosmotic stress-induced chromatin rewiring (Figure 1C). These findings demonstrate that hyperosmotic stress induces widespread rewiring of chromatin loops independent of cell type or specific osmoregulating stimuli.

### Sorbitol-induced loops are transient and characteristically distinct from pre-existing loops

**Fig. 2 |:**
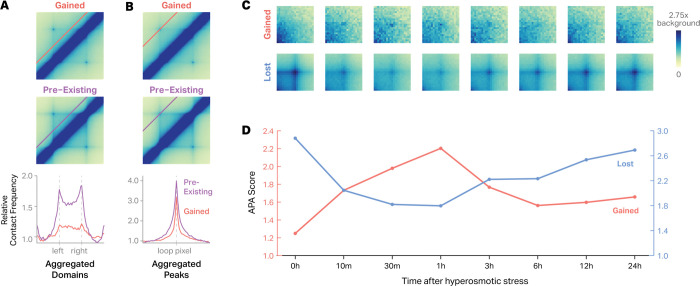
Sorbitol-induced loops are more punctate, form weaker chromatin domains, and peak at 1 hour of treatment. **A**, Aggregate Hi-C contact maps comparing gained (top) and pre-existing loops (middle). The red and purple lines on the plot denote the pixels represented in the line plots below. Line plots below show the relative contact frequency within the aggregated domain boundaries of gained (red line) and pre-existing domains (purple line). **B**, Aggregate Hi-C contact maps comparing gained (top) and pre-existing loops. Line plots below display the relative contact frequency profiles across aggregated chromatin loops before and after treatment. **C**, Aggregate Hi-C time-course analysis of chromatin loops following hyperosmotic stress. Heatmaps show aggregate contact patterns for gained loops (top row) and lost loops (bottom row) at eight time points: 0h, 10m, 30m, 1h, 3h, 6h, 12h, and 24h after sorbitol treatment. Color scale represents background-normalized contact frequency (0 to 2.75×), where background is defined as the median contact value from the top-left and bottom-right corners of each aggregate matrix. **D**, Quantitative analysis of chromatin loop enrichment over time. APA scores (y-axis) plotted against time after hyperosmotic stress treatment (x-axis: 0h to 24h). Red line shows gained loops, blue line shows lost loops.

To gain insight into the mechanisms driving sorbitol-induced loop formation, we characterized the contact frequency profiles of sorbitol-induced loops. To do so, we standardized loop dimensions to a uniform 150kb width and built aggregate Hi-C maps for 241 medium to long-range (>= 150kb) sorbitol-induced loops at 10kb resolution (Figure 2A, top). For comparison, we built aggregate Hi-C maps for a matched set of pre-existing loops (Figure 2A, middle), selected to have similar loop sizes and contact frequencies as sorbitol-induced loops. Sorbitol-induced loops were more punctate than pre-existing loops (Figure 2B, bottom left), even when controlling for loop strength and distance (Figure S2). The contact domains within sorbitol-induced loops also exhibited reduced contact frequency and had weaker ‘stripes’ along their borders. Sorbitol-induced loops are also longer on average than pre-existing loops (Figure S1C). The exact implications of these differences are unclear but are consistent with differences in the mechanisms of formation or maintenance compared to the pre-existing loops.

To understand the timing of formation and the persistence of sorbitol-induced loops, we quantified the dynamics of sorbitol-induced looping across an 8-point Hi-C (0h control, 10min, 30min, 1h, 3h, 6h, 12h, and 24h) time course. The cells were maintained in 200 mM sorbitol for the entire time course. Aggregate Peak Analysis (APA) revealed that sorbitol-induced loops are detectable by the earliest measured time point (10 min) after treatment and are most prominent after 1 hour. Sorbitol-induced loops are weakened but retain higher contact frequency than baseline even after 24 hours. Pre-existing loops were disrupted within 10 minutes of sorbitol treatment but began to re-establish as early as 3 hours after treatment (Figure 2C–D). Together, these results suggest that sorbitol triggers a distinct class of chromatin loops that are punctate, transient, and form weaker chromatin domains.

### CTCF, cohesin, and YAP1 are retained at sorbitol-induced loop anchors

**Fig. 3 |:**
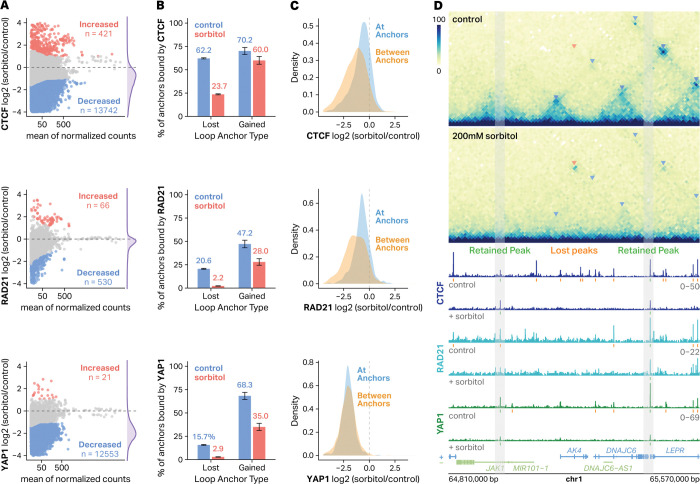
CTCF, cohesin, and YAP1 are retained at gained loop anchors following hyperosmotic stress. **A**, MA plots showing differential analysis of CTCF (top), RAD21 (middle), and YAP1 (bottom) binding (log_2_FC sorbitol/control) versus mean of normalized counts. Sites with significantly increased or decreased (DESeq2, padj < 0.05 for CTCF and YAP1; padj < 0.1 for RAD21) binding are highlighted in red and blue, respectively. **B**, Barplots depicting the percentage of loop anchors bound by CTCF (top), RAD21 (middle), and YAP1 (bottom). **C**, Density plots showing the distribution of changes in CTCF (top), RAD21 (middle), and YAP1 (bottom) occupancy of peaks at loop anchors (blue) compared to those within loop anchors (orange). **D**, Hi-C contact maps (10-kb resolution) at an example locus on chromosome 1 showing loop formation after 200 mM sorbitol treatment. Arrows denote gained (red) or lost (blue) loops. Corresponding CTCF and RAD21 CUT&Tag tracks are shown below. Retained (green) and lost (orange) peaks are denoted.

To determine whether stress-induced chromatin rewiring involves changes in architectural protein occupancy, we performed CTCF and RAD21 CUT&Tag in eGFP-YAP1 HEK293T cells that were either untreated or treated with sorbitol for 1 hour. Consistent with previous findings^19^, genome-wide differential analysis revealed that hyperosmotic stress led to global decreases in binding intensity (Figure 3A) with 13,742 CTCF peaks and 204 RAD21 peaks exhibiting reduced occupancy and only 421 CTCF peaks and 42 RAD21 peaks exhibiting increased occupancy (DESeq2, padj < 0.05 for CTCF and YAP1; padj < 0.1 for RAD21). Loops that were lost in response to sorbitol exhibited a precipitous loss in both CTCF and RAD21 occupancy which explains their decreased contact frequency (Figure 3B). In contrast, over 70% of gained loop anchors were bound by CTCF even before sorbitol treatment and largely remained bound after sorbitol treatment (Figure 3B). Similar trends were observed for RAD21 occupancy. Moreover, CTCF and RAD21 binding sites that were within gained loop anchors exhibited a more pronounced decrease in occupancy in response to sorbitol than binding sites at gained loop anchors (Figure 3C). These results are exemplified in Figure 3D in which lost CTCF and RAD21 binding is associated with lost loops and retained CTCF and RAD21 binding is observed at gained loop anchors. These results suggest that CTCF and RAD21 could play a critical role in sorbitol-induced looping.

Because we had previously observed phase separation-dependent nuclear localization and 3D clustering of YAP1 in response to sorbitol, we also mapped YAP1 occupancy using CUT&Tag. Surprisingly, we saw a global loss of YAP1 binding with 12,553 decreasing and only 21 increasing occupancy in response to sorbitol (DESeq2, padj < 0.05) (Figure 3A). Gained loop anchors were enriched for YAP1 binding sites (Figure 3B, bottom). Similarly to CTCF and RAD21, YAP1 occupied many (68.3%) gained loop anchors even in untreated cells, and was preferentially retained at gained loop anchors (Figure 3B). Interestingly, however, YAP1 occupancy showed strong depletion both at and between loop anchors (Figure 3C). Taken together, these results indicate that YAP1 could also play a role in sorbitol-induced looping.

We also mapped the occupancy profiles of histone H3K27 acetylation (H3K27ac) as it is a known marker of active enhancers and promoters and has previously been correlated with loop formation in the absence of CTCF^26^. Indeed, H3K27ac was enriched at sorbitol-induced loop anchors both before and in response to sorbitol treatment (Figure S4); however, no change was observed in H3K27ac at gained or lost loop anchors, nor was there a change in occupancy at or between loop anchors (Figure S4). While we cannot rule out H3K27ac’s role in sorbitol-induced loop formation, these results do not provide strong evidence of it.

### Sorbitol-induced loop formation require cohesin but not CTCF

**Fig. 4 |:**
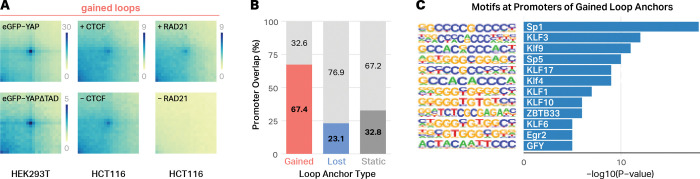
Sorbitol-induced chromatin loops require RAD21 and are enriched at promoters. **A**, Aggregate peak analysis (APA) plots showing the impact of sorbitol-induced looping after expression of a dominant negative YAP1, degradation of CTCF (middle), or degradation of RAD21 (right) . Acute loss of RAD21 markedly reduces sorbitol-induced loop signal, whereas CTCF depletion and expression of the YAP1 dominant negative have no discernable impact. **B**, Proportion of gained, lost, and static loop anchors that overlap annotated promoters. **C**, Enriched transcription factor binding motifs at promoters of gained loop anchors include SP1, KLF, and related promoter-associated transcription factors.

Due to their enrichment at sorbitol-induced loop anchors, we used perturbation experiments to directly test whether YAP1, RAD21, or CTCF were required for sorbitol-induced loop formation. First, we mapped 3D chromatin structure in cells expressing eGFP-YAP1ΔTAD in which the intrinsically disordered transcription activation domain (TAD) of YAP1 was deleted. This has previously been shown to function as a dominant negative, inhibiting phase separation of exogenous YAP1 and preventing both nuclear localization and condensate formation^20^. APA analysis of the resulting Hi-C data (Figure 4A) revealed no impact on sorbitol-induced looping, demonstrating that YAP1 is not responsible for sorbitol-induced loop formation. To test the requirement of canonical loop extrusion machinery, we acutely depleted CTCF or RAD21 in HCT116 cells using an auxin-inducible degron system, treated cells with 200 mM sorbitol for 1 hour, and mapped 3D chromatin contact frequencies using Hi-C. CTCF depletion also had no clear impact on sorbitol-induced loop formation (Figure 4A), suggesting that it is not required for loop formation under hyperosmotic stress. In contrast, RAD21 depletion completely eliminated sorbitol-induced loop enrichment (Figure 4A), demonstrating that cohesin-mediated extrusion is essential for sorbitol-induced loop formation.

Because cohesin was required but CTCF was dispensable, we next asked whether alternative genomic features—particularly regulatory elements—might preferentially anchor sorbitol-induced loops. Interestingly, sorbitol-induced loop anchors displayed a strong promoter bias: 67.4% overlapped annotated promoters, compared to 23.1% of lost anchors and 32.8% of static anchors (Figure 4B). Promoter regions underlying gained anchors were enriched for SP1 and multiple KLF transcription factor binding motifs, as well as additional GC-rich promoter-associated motifs (Figure 4C). These features align with prior work showing that promoter-bound transcription factors stabilize cohesin and organize long-range interactions in contexts where CTCF occupancy is reduced or insufficient for anchoring. Several transcription factor families, including SP/KLF factors, HO-1-associated SP1 in renal cells, and pluripotency regulators have been shown to recruit or stabilize cohesin at promoters and enhancers through mechanisms that do not require CTCF^27–30^.

These results point to a hyperosmotic stress-responsive looping mechanism in which cohesin continues to extrude but is preferentially anchored at promoter regions, providing an alternative stabilization strategy when CTCF is evicted from chromatin.

### Sorbitol-induced loop formation precedes expression of anchor genes

**Fig. 5 |:**
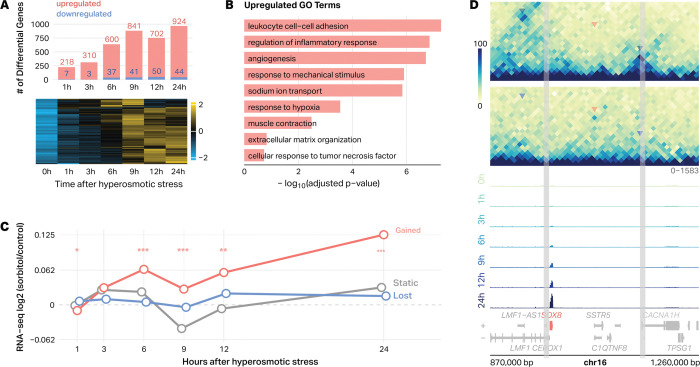
Sorbitol-induced loops are associated with transcriptional changes. **A**, Bar plot depicting differentially expressed genes (|log_2_ fold-change| > 2, padj < 0.05) in HEK293T cells treated with 200 mM sorbitol with labels to indicate number of up- and downregulated genes. Heatmap shows z-scored expression of 6,097 LRT-identified genes (DESeq2, LRT, p < 0.05), revealing transient, transcriptional programs across the time course. **B**, Gene Ontology (GO) enrichment of genes significantly upregulated or downregulated at any time point. Upregulated genes are enriched for inflammatory, cell–cell adhesion, and mechanical response pathways, while downregulated genes are enriched for RNA modification and translational control processes (ranked by −log_10_ adjusted p-value). **C**, Median transcriptional responses of genes at gained, static, and lost loop anchors. Promoters were assigned to loop classes based on overlap with sorbitol-induced, unchanged, or lost anchors. Lines show median RNA log_2_(sorbitol/control) across the time course. Genes at gained anchors exhibit stronger and sustained induction compared with static or lost groups. Asterisks denote time points where the mean log_2_FC for gained-anchor genes differs significantly from zero (*p < 0.05, **p < 0.01, ***p < 0.001; one-sample t-test, Benjamini-Hochberg adjusted). **D**, Example chromosome 16 locus showing a sorbitol-induced loop linked to transcriptional activation. Hi-C contact maps (10-kb) show loop gain, and RNA-seq tracks (0–24 h) show progressive SOX8 activation.

The strong promoter bias of sorbitol-induced loop anchors (Figure 4B) suggested that at least some of these structural changes might participate in the transcriptional response to hyperosmotic stress. To define this response, we performed a seven-point RNA-seq time course in HEK293T cells treated with 200 mM sorbitol (0, 1, 3, 6, 9, 12, and 24 hours). Relative to untreated controls, we observed a transcriptional program in which the number of significantly differentially expressed genes (absolute log_2_ fold change > 2, padj < 0.05) increased from a few hundred at 1 hour to over 900 by 24 hours, with the response overwhelmingly dominated by gene activation (Figure 5A). Likelihood-ratio testing identified 6,097 genes with significant time-dependent behavior, which were clustered based on their z-scored expression profiles (Figure 5A). Consistent with previous reports, we detect downstream of gene (DoG) transcription, although the effect is modest and restricted to select loci (Figure S5)^18,22^. Gene Ontology (GO) enrichment analysis revealed that upregulated genes were enriched for biological processes associated with cell-cell interactions and stress signaling, including leukocyte cell-cell adhesion, regulation of inflammatory response, angiogenesis, response to mechanical stimulus, and sodium ion transport (Figure 5B). Downregulated genes were not enriched for any specific biological processes.

These results confirm previous findings and demonstrate that hyperosmotic stress elicits a coordinated transcriptional program characterized by activation of stress-responsive and signaling-related pathways^9,10,18^. Moreover, only 27.2% of sorbitol-responsive genes localize to gained or lost loop anchors, whereas the remaining 72.8% fall outside these regions, indicating that loop remodeling accounts for only a subset of the transcriptional response. Genes whose promoters lie at sorbitol-induced loop anchors show increased expression in response to stress, with this upregulation emerging several hours after loop formation (Figure 5C). These changes are exemplified at the SOX8 locus (Figure 5D). Hi-C maps reveal the formation of a *de novo* loop connecting the SOX8 promoter and a distal region, coinciding with a progressive increase in SOX8 expression across the 24-hour time course (Figure 5D). Together, these analyses demonstrate that gained loops are preferentially anchored at promoters that become transcriptionally activated during the hyperosmotic stress response, linking stress-induced 3D chromatin structure to a subset of downstream gene expression programs.

## Discussion

Here, we characterized changes to 3D chromatin structure, protein-DNA interactions, and gene expression during the response of human cells to hyperosmotic stress. Our findings both agree with and extend those of previous studies. In agreement with prior work, we see evidence for loss of CTCF-mediated looping, run-on transcription, and large-scale changes in gene expression^18,19^. However, we extend those results by providing evidence of the formation of a new class of sorbitol-induced loops. These loops exhibit slight phenotypic differences to canonical CTCF-mediated loops, which could indicate different mechanisms of formation. One intriguing feature was that sorbitol-induced loops manifested as more punctate spots in Hi-C maps. These punctate structures are reminiscent of the punctate loops observed in Drosophila, which also do not involve CTCF^31^. Whether or not sorbitol-induced loops form via the same mechanisms as these Drosophila loops is unclear, as is why CTCF-driven loops are less punctate. These results offer another important data point that might help resolve such mysteries. We also extended previous knowledge by providing data on the timing and persistence of sorbitol-induced loop loss and formation. The timing of lost and gained loops showed a strong anticorrelation, which suggests that both may rely on the same underlying mechanisms. Indeed, our results are consistent with the loss of CTCF binding driving both looping phenotypes; however, proving this mechanism will require further investigation.

This project was largely motivated by our previous work that suggested YAP1 might mediate 3D chromatin changes in response to hyperosmotic stress; however, the results from this study only partially agree. In our previous work, we found that in response to sorbitol, YAP1 enters the nucleus and forms condensates that colocalize with hubs of open chromatin. In the current study, we did observe large-scale changes in 3D chromatin structure, including the formation of new loops whose anchors were enriched for YAP1. Surprisingly, however, the expression of a dominant negative form of YAP1, which we previously found inhibits nuclear localization and condensate formation (of both exogenous and endogenous YAP1), did not inhibit the formation of sorbitol-induced chromatin loops. This is strong evidence that YAP1 is not required for sorbitol-induced loop formation. One possible explanation for the discrepancy between our current Hi-C-based results and our previous 3D ATAC-PALM results is that 3D ATAC-PALM and Hi-C probe chromatin organization on fundamentally different spatial scales. Hi-C requires that two regions be within a crosslinking radius, which some estimate to be as small as tens of nanometers^32–35^. In contrast, even with very high resolution analysis, 3D ATAC-PALM can only resolve colocalization events down to a radius of 100 to 400nm^20,36^. Therefore, the type of clustering observed by 3D ATAC-PALM may not be sufficient for detection by Hi-C. Further work will be required to disentangle the precise role of YAP1 in sorbitol-induced 3D chromatin changes, but the existing data are consistent with YAP1 playing a role in large-scale changes to nuclear localization but no role in changes to chromatin looping.

Our findings extend a growing body of work demonstrating that transcription factor-decorated regulatory elements can stabilize cohesin and organize 3D chromatin structure independently of CTCF. Previous studies by Bryan et al. showed that common transcription factors and chromatin regulators, including SP1 and WDR5, are required to maintain cohesin at promoters and enhancers genome-wide^27^, while locus-specific studies revealed that Sp1 and Klf4 directly organize long-range chromatin interactions at stress-responsive and pluripotency loci, respectively^28,29^. More recently, Georgiades et al. demonstrated that active enhancers are sufficient to recruit cohesin and generate new cell type-specific chromatin domains *in vivo,* concordant with our findings that H3K27ac is enriched at sorbitol-induced loop anchors^30^. Our data place hyperosmotic stress within this same mechanistic framework where despite global loss of cohesin and CTCF, sorbitol-induced loops preferentially form at SP/KLF motifs at promoters and require RAD21, but not CTCF. Together, these results suggest that under hyperosmotic stress, transcription factor-decorated promoters act as alternative cohesin anchoring sites, enabling rapid rewiring of 3D chromatin structure during the hyperosmotic stress response.

Genes at the anchors of sorbitol-induced loops exhibit a modest but significant increase in expression in response to sorbitol. Importantly, gene expression does not significantly increase until several hours after loop formation. In fact, gene expression peaks at 24 hours at which time the sorbitol-induced loops have largely disappeared. This timing argues against a model in which loop formation is merely a passive consequence of transcriptional activation. Instead, the results are consistent with the loops playing a causal role in gene activation, though would require further investigation to confirm. It is important to note that the changes in gene expression and sorbitol-induced anchors are modest and variable. Moreover, the majority of sorbitol-responsive genes are not found at gained or lost loop anchors. Taken together, this suggests that loops are just one of many regulatory mechanisms that control the transcriptional response to hyperosmotic stress.

There are several limitations to this study. First, our conclusions are derived from bulk Hi-C, CUT&Tag, and RNA-seq data, which average over heterogeneous cell populations. Individual cells likely exhibit varied degrees and trajectories of chromatin collapse, loop gain, and transcriptional response that are not resolved in our current datasets. Second, crosslinking-based assays provide static snapshots of nuclear organization, limiting our ability to infer causality or fine-grained temporal order between specific looping events and changes in transcription beyond the coarse time resolution of our time courses. Third, our mechanistic perturbations rely on engineered cell lines (eGFP-YAP1 and eGFP-YAP1ΔTAD HEK293T cells; auxin-inducible CTCF and RAD21 degrons in HCT116 cells), which may differ in chromatin context and osmoadaptive pathways from primary renal, immune, or other physiologically relevant cell types. Finally, we focused on a subset of architectural and regulatory factors; additional contributors—including other cohesin regulators, chromatin remodelers, or nuclear envelope components—may participate in shaping the stress-induced 3D genome but were not directly examined here.

Together, our results reveal that hyperosmotic stress triggers a rapid, global, and reversible rewiring of 3D chromatin structure characterized by the collapse of canonical CTCF-mediated loops and the emergence of a distinct class of CTCF-independent, cohesin-dependent sorbitol-induced loops. These findings redefine how physical genome organization adapts under acute environmental stress and support accessible promoters as alternative architectural scaffolds during periods of widespread chromatin destabilization. More broadly, this study highlights the remarkable plasticity of the 3D genome and suggests that environmental stress can transiently reshape nuclear architecture through mechanisms that are fundamentally distinct from those operating during homeostasis.

## METHODS

### Experimental model and subject details

#### Cell Lines

HEK293T eGFP-YAP1, HEK293T eGFP-YAP1dTAD (gifts from the D. Cai Lab) and HEK293T (ATCC CRL-3216) cells were cultured at 37 °C and 5% CO_2_ in DMEM supplemented with 10% fetal bovine serum (FBS; Gibco) and 100 U ml-1 penicillin/streptomycin (Corning). HCT116 (ATCC CCL-247), HCT116-RAD21-mAID2, and HCT116-CTCF-mAID2 (obtained from M. Kanemaki Lab) cells were cultured at 37 °C and 5% CO_2_ in McCoy’s 5A medium supplemented with 10% FBS (Gibco) and 100 U ml-1 penicillin/streptomycin (Corning). Unless noted, cells were grown on 10-cm Corning plates.

#### Sorbitol Treatments

For high-depth Hi-C experiments, wild-type HEK293T, eGFP-YAP1 HEK293T, and wild-type HCT116 cells were serum-starved for 1 h and treated with control serum-free media or 0.2 M sorbitol (MP Biomedicals, cat #194742) in serum-free media for 1 h, as described by Hong et al^37^. For Hi-C time course experiments, wild-type HEK293T cells were serum-starved for 1 h and treated with control serum-free media for 1 h or 0.2 M sorbitol for 10 m, 30 m, 1 h, 3 h, 6 h, 12 h, and 24 h. For low-depth Hi-C experiments, HEK293T eGFP-YAP1dTAD cells were serum-starved for 1 h and treated with 0.2 M sorbitol for 1 h. For RNA-seq time course experiments, wild-type HEK293T cells were serum-starved for 1 h and treated with control serum-free media for 1 h or 0.2 M sorbitol for 1 h, 3 h, 6 h, 9 h, 12 h, and 24 h.

#### Auxin Treatments

HCT116-RAD21-mAID2 and HCT116-CTCF-mAID2 cells were serum-starved and treated with 1 μM 5-phenyl-indole-3-acetic acid (5-Ph-IAA) (BioAcademia, Japan, #30–003) for 6 h, adapted from Yesbolatova et al^38^. Cells were then treated with 0.2 M sorbitol in serum-free media with or without 1 μM auxin (5-Ph-IAA) for 1 h.

#### Hi-C Library Generation

Cells were treated in serum-free, sorbitol-containing DMEM as described above, then crosslinked in 1% formaldehyde (Fisher Scientific, Cat# 28908) for 10 min at room temperature and quenched with 0.2 M glycine (Fisher Scientific, Cat# 07–678-003) for 5 min. Cells were washed twice with ice-cold PBS, pelleted (~5 × 10^6^ cells/pellet), snap-frozen in liquid nitrogen, and stored at −80 °C. In situ Hi-C libraries were prepared following the protocol of Rao et al. 2014^35^, including nuclei isolation, MboI digestion, biotin-dATP fill-in, proximity ligation, crosslink reversal, and DNA purification^35^. Genomic DNA was sheared to 300–500 bp using a Covaris LE220 (DF 20, PIP 100, 200 cycles/burst, 80 s), followed by AMPure XP size selection. Biotinylated fragments were captured with streptavidin beads, and all subsequent steps (end-repair, A-tailing, and adaptor ligation) were performed on-bead using TruSeq Nano adapters. Libraries were amplified by PCR (7–10 cycles), quality-checked (Qubit dsDNA HS and Agilent TapeStation D1000), pooled to a final concentration of 10 nM, and sequenced in paired-end 150 bp mode on an Illumina NovaSeq X Plus using an S4 flow cell (2 × 150 bp).

#### RNA-seq Library Generation

Total RNA was extracted using the QIAGEN RNeasy Mini Kit (Cat. #74104/74106) with on-column RNase-free DNase I (Cat. #79254) per manufacturer’s protocol. RNA integrity was assessed by Qubit and Agilent TapeStation. Extracted RNA was shipped on dry ice to Azenta Life Sciences, where total RNA rRNA depletion and stranded RNA-seq library preparation (KAPA RNA HyperPrep with RiboErase HMR) were performed. Libraries were sequenced paired-end 150 bp on an Illumina NovaSeq X-series instrument, targeting ~30 million read pairs per sample.

#### CUT&Tag Library Generation

CUT&Tag was performed as described by Kaya-Okur et al. with minor modifications^39^. Briefly, ~1 × 10^5^ cells per sample were harvested and supplemented with 5% GFP-H2B-expressing NIH3T3 cells for spike-in normalization. Cells were washed in Wash Buffer (HEPES, NaCl, spermidine, protease inhibitors) and bound to concanavalin-A-coated magnetic beads (10 μL/sample; activated in Bead Activation Buffer) for 10 min at room temperature (RT). Bead-bound cells were resuspended in Digitonin 150 Buffer (±2 mM EDTA) and incubated with primary antibodies overnight at 4 °C. Primary antibodies were used at 1:50 and included anti-GFP to target eGFP-YAP1 (Abcam, #ab290), anti-CTCF (Cell Signaling Technology, #3418S), anti-RAD21 (Abcam, #ab217678), and anti-H3K27ac (Abcam, #ab4729). After washing, cells were incubated with secondary antibody (1:50, 30 min, RT), followed by the pre-loaded pA-Tn5 adapter complex (1:200 in Digitonin 300 Buffer) for 1 h at RT. Unbound enzyme was removed and tagmentation was initiated in Digitonin 300 Buffer supplemented with 10 mM MgCl_2_ (37 °C, 1 h). DNA was released with SDS Release Buffer (58 °C, 1 h), quenched with SDS Quench Buffer, and purified using AMPure XP beads. Indexed libraries were generated using universal i5 and barcoded i7 primers, PCR-amplified with 2× master mix, cleaned with 0.9× AMPure XP, eluted in 10 mM Tris-HCl (pH 8.0), and sequenced (PE75) on an Illumina NextSeq 500 or equivalent platform.

### Quantification and statistical analysis

#### Hi-C Processing and Loop Detection

Hi-C data were processed using a custom pipeline called *dietJuicer* (Snakemake workflow; https://github.com/PhanstielLab/dietJuicer), derived from Juicer^40^. Reads were aligned to the hg38 genome using bwa mem (v0.7.17) and filtered to valid pairs (MAPQ ≥ 30)^41^. In situ Hi-C libraries were generated using MboI restriction digestion^35^. Hi-C contact matrices were generated at 5-kb and 10-kb resolutions and balanced using SCALE normalization for visualization and APA plots. Chromatin loops were identified using SIP (v1.6.1) (https://github.com/PouletAxel/SIP) with parameters -g 1 -t 2000 -fdr 0.05 (using -isDroso false), and loop anchors were lifted to a 10-kb grid and merged across replicates using DBSCAN clustering (ε = 20 kb; Manhattan distance)^31,42,43^. For differential loop analysis, raw observed loop pixel counts were extracted at 10-kb resolution directly from per-sample .hic files using strawr (v0.0.9) as implemented in mariner, ensuring no matrix normalization was applied (i.e., norm = NONE, matrix = observed)^44^. SCALE-normalized matrices were used exclusively for visualization and aggregate peak analysis.

#### Differential Loop Analysis

Loop counts were modeled in DESeq2 with the design ~ Replicate + Treatment^45^. Because hyperosmotic stress induces global changes in chromatin interaction frequencies, we expected widespread differences in total loop counts between conditions. To avoid normalization procedures that assume global count stability and could therefore mask true genome-wide remodeling, size factors were fixed to 1 (sizeFactors(dds) <- 1), ensuring loops were compared on an unscaled basis. Wald tests were used for contrasts, and log_2_ fold-changes were shrunk using apeglm^46^. Unless stated otherwise, gained loops were defined as padj < 0.1 with log_2_FC > 0, and lost loops as padj < 0.1 with log_2_FC < 0. For QC and visualization, variance-stabilized counts were converted to Z-scores and clustered by k-means.

#### Aggregate Hi-C analysis

Aggregate loop and domain analyses were performed using mariner (v1.5.0) and strawr (v0.0.9). KR- or VC_SQRT-normalized Hi-C contact matrices were extracted at 10-kb resolution. Loops with inter-chromosomal anchors or insufficient signal (row/column sums < 1) were excluded. Loop-centric aggregates were generated by centering windows on loop pixels with a ±0.5-loop-width buffer. For domain-centric aggregates, windows were scaled to loop span and subsequently resized to a 100 × 100 matrix using bilinear interpolation. Each window was normalized by total contact count prior to averaging across loops.

Loop strength was quantified as enrichment of the central pixel (or central 3×3 window) relative to a local background, defined either as the median of flanking ring bins or corner regions (for APA-style summaries). One-dimensional enrichment profiles were derived by extracting diagonal traces and normalizing to the corresponding background estimate.

For comparisons to size- and contact-matched null loop sets, we used the matchRanges function from the nulllranges package (v1.14.0) to select control loops matched on log(loop size) and log(aggregated contact frequency) using stratified sampling without replacement.

#### Hi-C time course analysis

We analyzed chromatin loop dynamics in wild-type HEK293T cells (0 h, 10 min, 30 min, 1 h, 3 h, 6 h, 12 h, 24 h; control and sorbitol). Differentially gained and lost loops (DESeq2) were used as input, and subsampled Hi-C maps were processed in R with mariner (v1.5.0) and visualized with plotgardener (v1.5.0)^44,47^.

Aggregate peak analysis (APA) was performed at 10-kb resolution using ±10-bin windows, with observed counts extracted by pullHicMatrices and normalized by loop number. Loop enrichment was calculated with mariner’s calcLoopEnrichment, which defines the foreground as the loop center and background as the top-left and bottom-right corners. Enrichment was computed as median(foreground + 1)/median(background + 1), controlling for distance-dependent decay.

#### Matched chromatin loop sets

To compare gained loops against size/contact-matched controls, we used the matchRanges function from the nullranges package (v1.14.0) (stratified, no replacement) with covariates log(loop size) and log(aggregated contact frequency). Aggregated contact frequency combined sorbitol and control counts per loop; zero values were treated as missing prior to log transformation.

#### RNA-seq processing and quantification

RNA-seq reads were processed using the bagPipes Snakemake workflow (https://github.com/PhanstielLab/bagPipes/tree/v2.0.0). Adapter trimming was performed with Trim Galore, and read quality was assessed using FastQC and MultiQC^48–50^. Transcript quantification was carried out using Salmon (v1.10.0; --validateMappings --seqBias --gcBias -posBias) against GENCODE hg38 reference indices^51^. Quantification files were imported into R using tximeta, and counts were summarized to the gene level with tximport^52,53^. Genes with <10 counts in fewer than 4 samples were excluded. All samples showed RIN > 9.8 and passed quality control.

#### Differential gene analysis

Transcript-level Salmon^51^ quantifications were imported with tximeta^53^ and summarized to gene-level counts. Genes with low expression were excluded by requiring at least 50 raw counts in ≥ 4 samples prior to modeling. Gene-level counts were analyzed in DESeq2^45^ with the design ~ Bio_Rep + Time, treating biological replicate and timepoint as fixed effects. To identify genes whose expression changed over the time course, we fit a likelihood ratio test (LRT) model comparing the full design (~ Bio_Rep + Time) to a reduced model lacking the time term (~ Bio_Rep), and defined genes with FDR-adjusted p < 0.05 as exhibiting significant time-dependent variation. Timepoint-specific responses were then quantified by extracting the coefficients for each Time_Xh_vs_0h comparison from the same model; unless otherwise stated, genes were classified as differentially expressed at a given timepoint if padj < 0.05 and |log_2_FC| > 2 relative to 0h. For downstream visualization, variance-stabilized counts were averaged across biological replicates, Z-scored per gene, and subjected to k-means clustering (k = 3) to distinguish early/transient from late/sustained transcriptional programs. The stacked barplot in Figure 5 reports the number of upregulated (log_2_FC > 2) and downregulated (log_2_FC < −2) genes at each timepoint, and the accompanying heatmap displays the clustered, Z-scored temporal expression profiles of these genes over the 0–24 h sorbitol time course.

#### CUT&Tag processing and peak calling

CUT&Tag sequencing reads were processed using a Snakemake-based ChIP/CUT&Tag workflow derived from bagPipes (https://github.com/PhanstielLab/bagPipes/tree/v2.0.0). Adapter trimming and initial quality assessment were performed using Trim Galore and FastQC^48,49^. Reads were aligned to the hg38 reference genome using BWA-MEM (v0.7.17), and resulting alignments were sorted and indexed using samtools^41,54^. PCR duplicates were removed using Picard *MarkDuplicates* (REMOVE_DUPLICATES=true), and reads overlapping ENCODE hg38 blacklist regions were removed using bedtools (v2.31.1)^55,56^.

For peak calling, replicate BAM files were merged by condition and peaks were identified using MACS2 (v2.2.6; callpeak -f BAMPE -g hs --keep-dup 1 -q 0.01)^57^. Peaks were annotated using HOMER^58^. A unified peak set was generated by merging peak coordinates across samples using bedtools merge, and fragment counts per sample were quantified using bedtools multicov to generate a peak-by-sample count matrix for differential analysis.

Spike-in normalization was evaluated at the analysis stage. In experiments including NIH3T3 spike-in nuclei, spike-in-derived fragments were used to verify normalization stability across samples; however, no spike-in scaling factor was applied directly to read counts.

#### Differential CUT&Tag Analysis

Differential peak analysis was performed separately for CTCF, RAD21, H3K27ac, and YAP1. CUT&Tag datasets using DESeq2 with the design ~ Replicate + Treatment. Because hyperosmotic stress alters global chromatin occupancy, we expected widespread differences in total peak counts between conditions; to avoid normalization procedures that assume global count stability and could obscure true genome-wide changes, size factors were fixed to 1 (sizeFactors(dds) <- 1), and peaks were compared on an unscaled basis. Peaks with low counts were removed prior to modeling by requiring a mean normalized count ≥ 15 across all samples. Wald tests were used to assess treatment effects, and log_2_ fold-changes were shrunk using apeglm. For CTCF and YAP1, increased peaks were defined as padj < 0.05 with log_2_FC > 1 and decreased peaks as padj < 0.05 with log_2_FC < −1. For RAD21, a slightly relaxed significance threshold (padj < 0.1) was used to account for lower signal-to-noise ratios observed in this dataset. For QC and visualization, variance-stabilizing or rlog-transformed counts were used for principal component analysis and sample-sample correlation heatmaps, and MA plots were inspected to confirm model behavior.

#### Re-analysis of Publicly Available Data

Public Hi-C data from Amat et al. 2019 were downloaded from NCBI Gene Expression Omnibus (GEO) under accession number GSE111904 and processed through the same dietJuicer in-house Snakemake workflow against hg38, using identical alignment (bwa mem v0.7.17), filtering (MAPQ ≥ 30), SCALE normalization, SIP loop-calling parameters, and downstream counting with mariner (v1.5.0)^31,40,41,44^. When re-analyzing external RNA-seq or CUT&Tag datasets, we used the bagPipes workflows with the same tool versions and thresholds described above so that comparisons to our datasets were methodologically harmonized.

### Declaration of generative AI and AI-assisted technologies

During the preparation of this work the author(s) used ChatGPT in order to identify weaknesses, correct grammar, and brainstorm titles. After using this tool/service, the author(s) reviewed and edited the content as needed and take(s) full responsibility for the content of the published article.

## Supplementary Material

Supplement 1

## Data Availability

All raw and processed sequencing data generated in this study have been submitted to the NCBI Gene Expression Omnibus (GEO; https://www.ncbi.nlm.nih.gov/geo/). The Hi-C data are available under accession number GSE310051. The Hi-C data for HCT116-RAD21-mAID2 and HCT116-CTCF-mAID2 cells are available under accession number GSE312288. The RNA-seq data are available under accession number GSE310049. The CUT&Tag data are available under accession number GSE310047. The code to process and analyze these data is available on GitHub (https://github.com/jpflores-13/STRS).
