# Supplementary material for: Stress-induced loss of CTCF reveals an alternative, promoter-based mode of cohesin looping
Source: bioRxiv. 2025 Dec 22:2025.12.19.695003. Preprint. [Version 1] doi: 10.64898/2025.12.19.695003 (PMC12767365; doi:10.64898/2025.12.19.695003)
Supplement: Supplement 1 [file NIHPP2025.12.19.695003v1-supplement-1.pdf]

bioRxiv preprint doi: https://doi.org/10.64898/2025.12.19.695003; this version posted December 22, 2025. The copyright holder for this preprint (which was not certified by peer review) is the author/funder, who has granted bioRxiv a license to display the preprint in perpetuity. It is made available under aCC-BY 4.0 International license.

Supplementary Figures

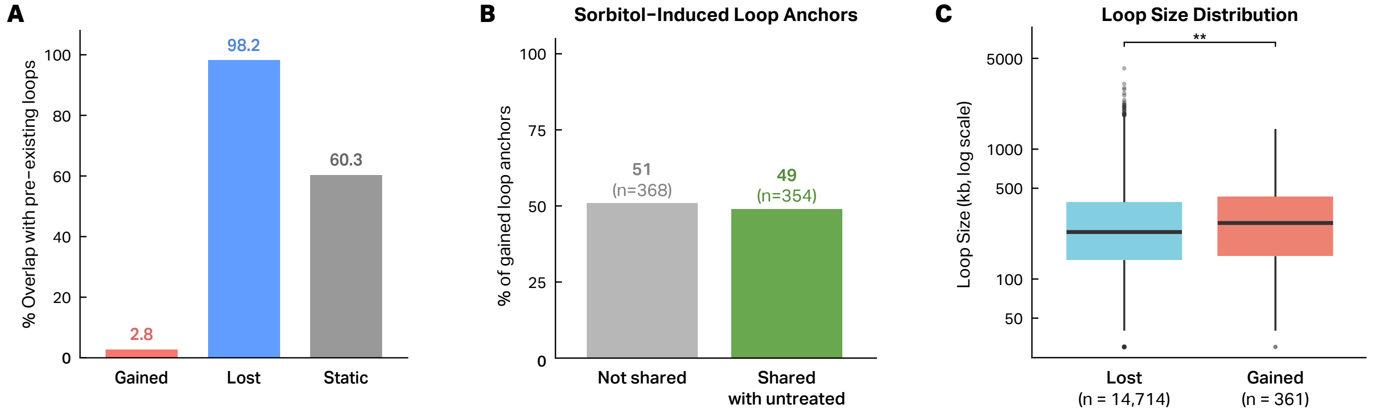

Fig. S1 | Hyperosmotic stress induces predominantly de novo looping that frequently reuses pre-existing anchor sites and favors long-range interactions. **A**, Percent overlap between differential loop categories and loop calls in untreated cells. Only 2.8% of sorbitol-induced (gained) loops overlap pre-existing loops, indicating that ~97% of gained loops are newly formed rather than strengthened pre-existing interactions. Lost and static loops overlap with untreated loops at 98.2% and 60.3%, respectively. **B**, Proportion of sorbitol-induced loop anchors that coincide with loop anchor sites present in untreated cells. 49% of gained loop anchors (n = 354/722) overlap untreated anchor positions, whereas 51% are unique to sorbitol-treated cells. **C**, Size (kb) of lost and gained loops. Gained loops are significantly longer than lost loops (Wilcoxon rank-sum test; ****p < 1×10^{-4}, p < 0.01), indicating preferential formation of long-range chromatin contacts under hyperosmotic stress.

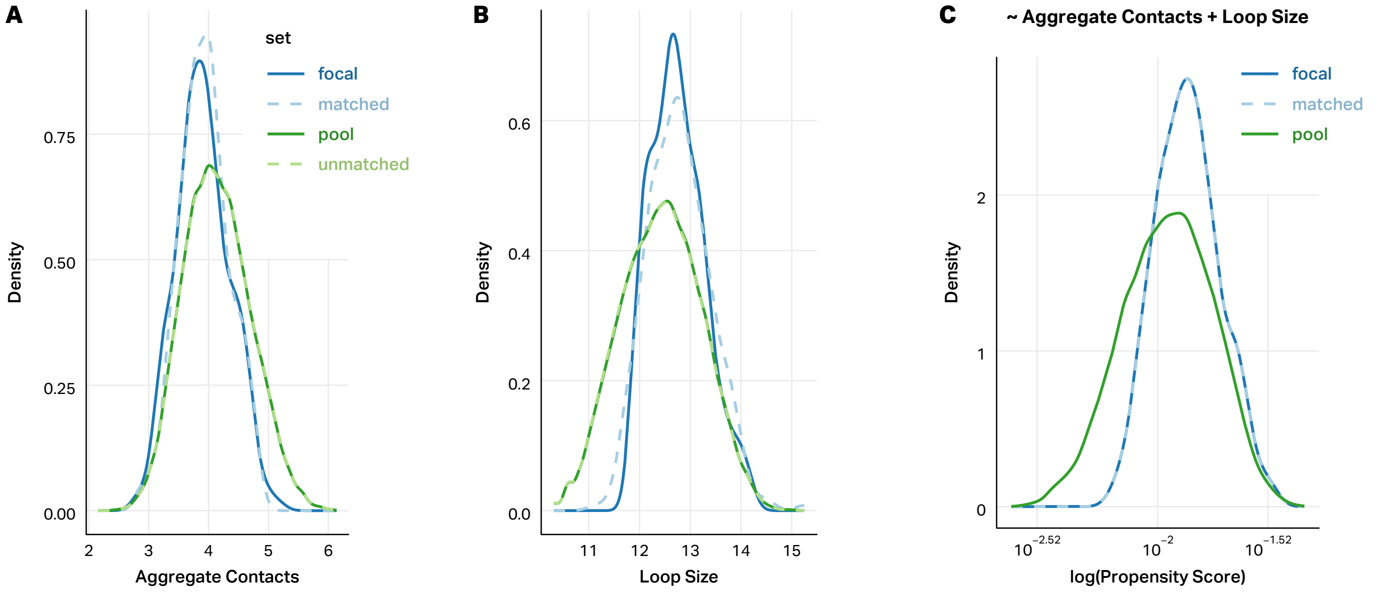

Fig. S2 | Covariate matching controls for loop size and interaction frequency in gained loop comparisons.. **A**, Density distributions of aggregated Hi-C contact counts at loop anchors for focal gained loops (solid blue), the matched null set (dashed blue), and the full pool of candidate control loops (solid/dashed green). Matching reduces differences in contact intensity between the focal and control sets. **B**, Density distributions of loop size (log-transformed genomic span) for the same loop sets. The matched null set closely recapitulates the size distribution of focal gained loops. **C**, Propensity score distributions for focal, pool, and matched loops, demonstrating effective balancing of the joint covariate structure (aggregated contacts and loop size). Together, these diagnostics confirm that downstream comparisons of chromatin features are not confounded by differences in contact frequency or loop span.

bioRxiv preprint doi: https://doi.org/10.64898/2025.12.19.695003; this version posted December 22, 2025. The copyright holder for this preprint (which was not certified by peer review) is the author/funder, who has granted bioRxiv a license to display the preprint in perpetuity. It is made available under aCC-BY 4.0 International license.

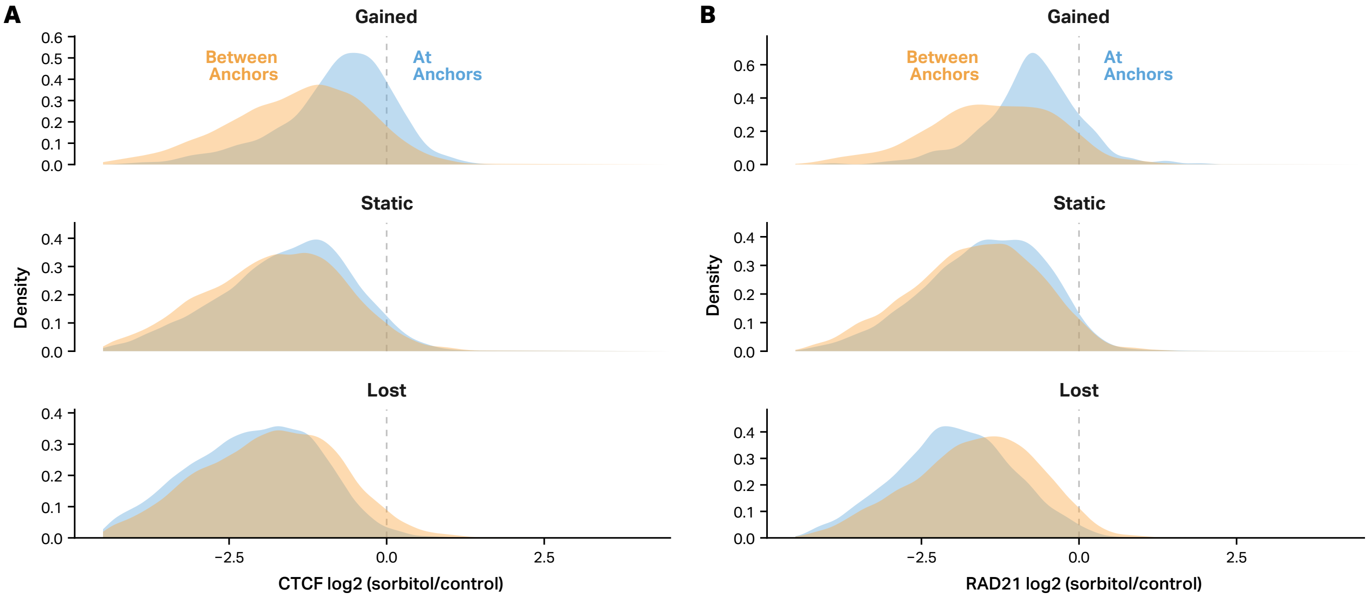

Fig. S3 | CTCF and cohesin binding is selectively retained at anchors of sorbitol-induced chromatin loops. **A**, Density distributions of CTCF $\log_2$ fold–change values (sorbitol/control) at loop anchors (blue) versus the genomic regions between loop anchors (orange), stratified by loop class (static, lost, and gained). Gained loop anchors show a right-shifted distribution relative to between-anchor regions, indicating preferential retention of CTCF at newly formed loop anchors following hyperosmotic stress. Static and lost loops do not show this pattern. **B**, Equivalent density distributions for RAD21 reveal a similar preferential retention of cohesin at gained loop anchors relative to between-anchor regions, while static and lost anchors exhibit symmetric or left-shifted distributions. Together, these analyses indicate that CTCF and cohesin stabilization at loop anchors is a distinguishing feature of sorbitol-induced loop formation.

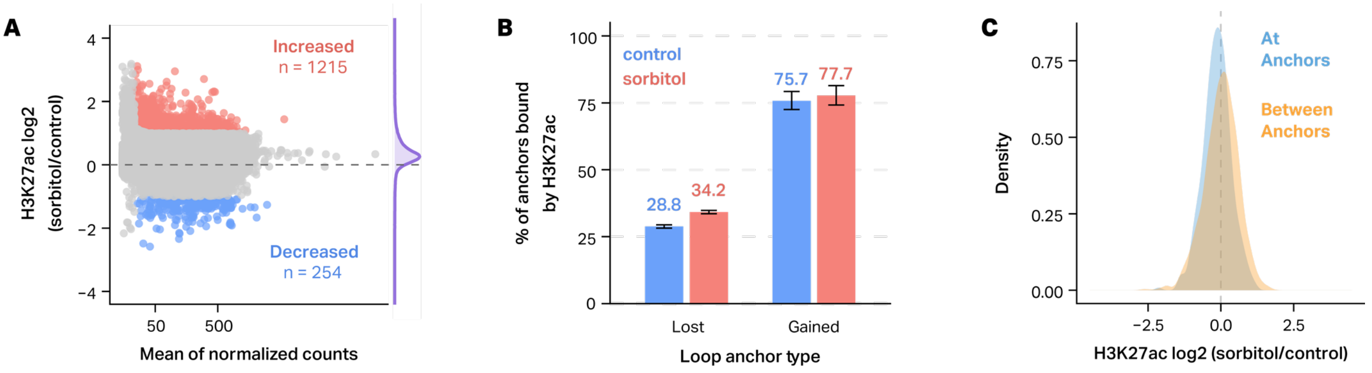

Fig. S4 | Hyperosmotic stress differentially modulates H3K27ac binding at loop anchors. **A**, MA plots showing differential CUT&Tag signal for H3K27ac in sorbitol-treated versus control HEK293T cells. The x-axis denotes the mean of normalized counts and the y-axis shows $\log_2$ occupancy (sorbitol/control); peaks with significantly increased or decreased signal (DESeq2, padj < 0.05) are highlighted in red and blue, respectively, with the number of sites indicated. **B**, Percentage of lost and gained loop anchors that are bound by H3K27ac in control (blue) and sorbitol-treated (red) cells. Gained loop anchors are frequently marked by H3K27ac both before and after treatment. **C**, Density plots of H3K27ac occupancy changes at gained loop anchors, comparing peaks at loop anchors (blue) versus the regions between anchors (orange).

bioRxiv preprint doi: https://doi.org/10.64898/2025.12.19.695003; this version posted December 22, 2025. The copyright holder for this preprint (which was not certified by peer review) is the author/funder, who has granted bioRxiv a license to display the preprint in perpetuity. It is made available under aCC-BY 4.0 International license.

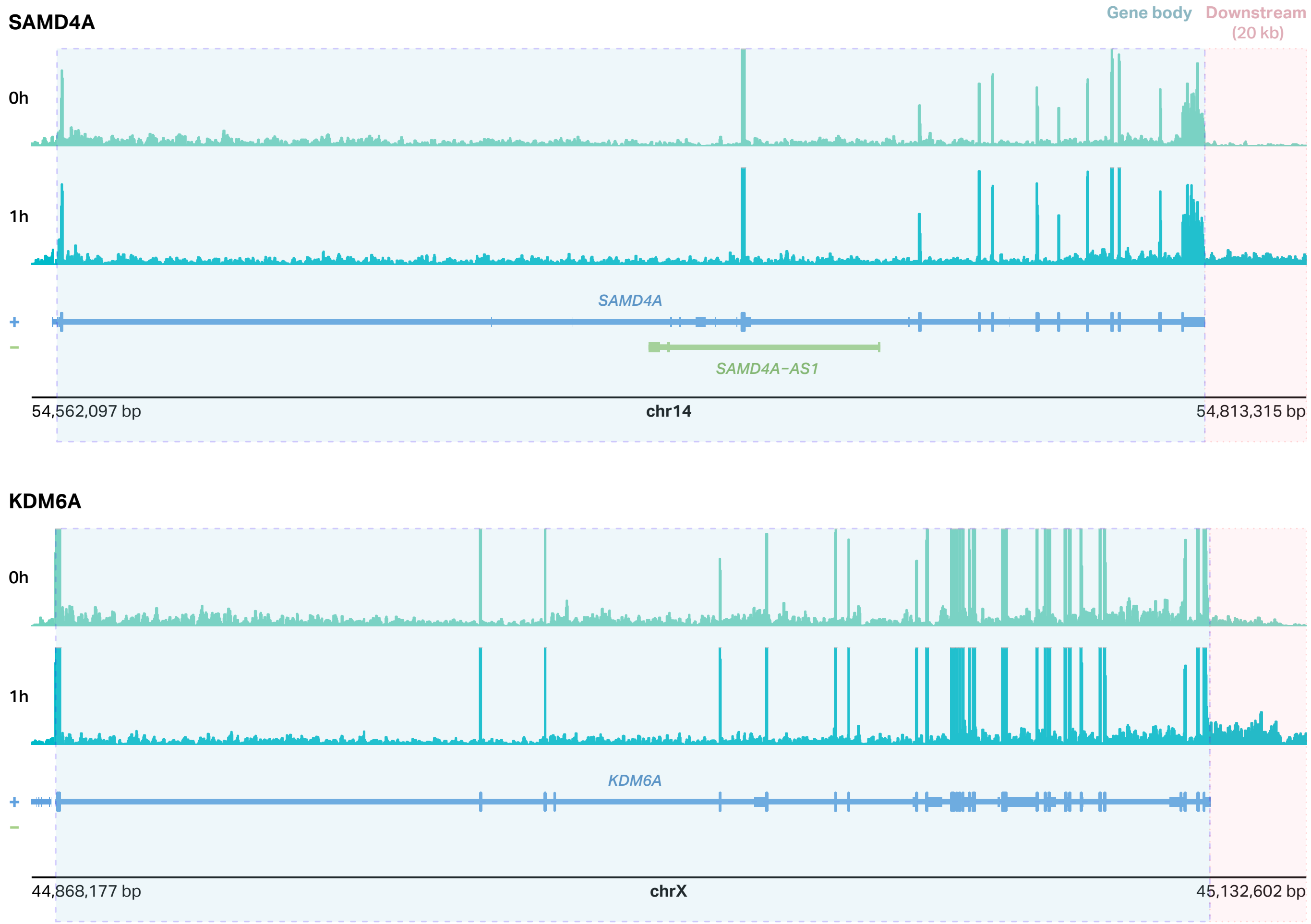

Fig. S5 | Hyperosmotic stress induces downstream-of-gene (DoG) transcription at select loci. Genome browser views of RNA-seq signal at the SAMD4A (top) and KDM6A (bottom) loci in HEK293T cells before (0 h) and after 1 h of 200 mM sorbitol treatment. The annotated gene body is highlighted in blue and the 20-kb downstream region used for DoG detection is shaded in pink.
